# The Intriguing Dual Lattices of the Myosin Filaments in Vertebrate Striated Muscles: Evolution and Advantage

**DOI:** 10.3390/biology3040846

**Published:** 2014-12-03

**Authors:** Pradeep K. Luther, John M. Squire

**Affiliations:** 1 Molecular Medicine Section, National Heart and Lung Institute, Faculty of Medicine, Imperial College London, London SW7 2AZ, UK; 2 Muscle Contraction Group, School of Physiology & Pharmacology, University of Bristol, Bristol BS8 1TD, UK; E-Mail: j.m.squire@bristol.ac.uk; 3 Department of Surgery and Cancer, Faculty of Medicine, Imperial College London, London SW7 2AZ, UK

**Keywords:** vertebrate striated muscle, electron microscopy, X-ray diffraction, myosin filament lattice, thick filament, myosin filament, M-band, bare-region

## Abstract

Myosin filaments in vertebrate striated muscle have a long roughly cylindrical backbone with cross-bridge projections on the surfaces of both halves except for a short central bare zone. In the middle of this central region the filaments are cross-linked by the M-band which holds them in a well-defined hexagonal lattice in the muscle A-band. During muscular contraction the M-band-defined rotation of the myosin filaments around their long axes influences the interactions that the cross-bridges can make with the neighbouring actin filaments. We can visualise this filament rotation by electron microscopy of thin cross-sections in the bare-region immediately adjacent to the M-band where the filament profiles are distinctly triangular. In the muscles of teleost fishes, the thick filament triangular profiles have a single orientation giving what we call the simple lattice. In other vertebrates, for example all the tetrapods, the thick filaments have one of two orientations where the triangles point in opposite directions (they are rotated by 60° or 180°) according to set rules. Such a distribution cannot be developed in an ordered fashion across a large 2D lattice, but there are small domains of superlattice such that the next-nearest neighbouring thick filaments often have the same orientation. We believe that this difference in the lattice forms can lead to different contractile behaviours. Here we provide a historical review, and when appropriate cite recent work related to the emergence of the simple and superlattice forms by examining the muscles of several species ranging back to primitive vertebrates and we discuss the functional differences that the two lattice forms may have.

## 1. Introduction: The Sarcomere, A-Band, Simple Lattice and Superlattice

**Figure 1:**
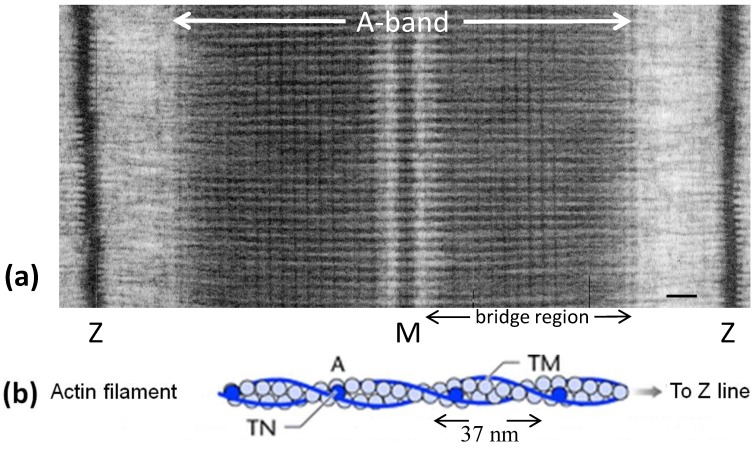

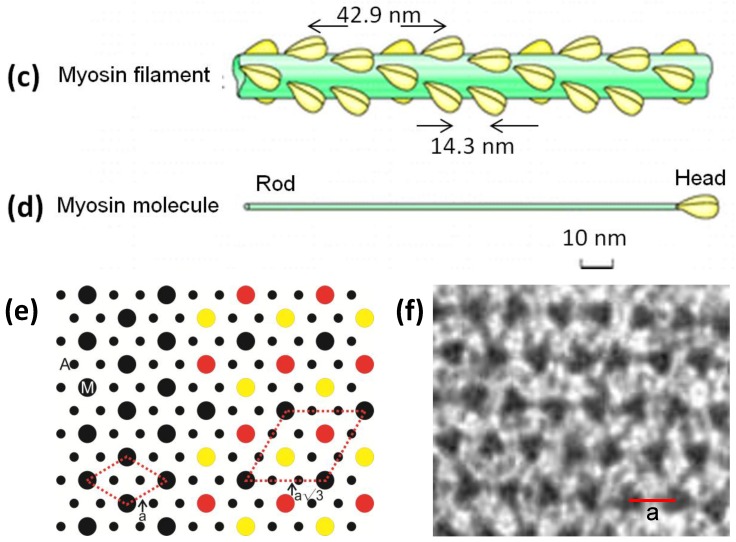
(**a**) Electron micrograph of a longitudinal section from frog sartorius muscle showing the Z-bands (Z), A-band (central dark region containing the M-band (M), and flanking bridge regions). Sarcomere length (Z to Z) is about 2.2 µm. Scale bar: 100 nm. (**b**) Actin filament shown schematically; A actin monomers; TM tropomyosin; TN troponin. (**c**) Schematic diagram of part of the bridge region of a myosin filament in vertebrate striated muscles. The backbone (green) consists mainly of myosin rods, and the myosin heads (yellow) are arranged in a roughly helical lattice on the backbone surface. Crowns of heads are separated by 14.3 nm, and the structure repeats after 42.9 nm. (**d**) A myosin molecule composed of a rod and two heads. (**e**) Hexagonal lattice of filaments showing unit cells (red dotted lines) of the basic (simple) lattice (left) depicted in black and superlattice (right) depicted in red, yellow and black; each colour represents separate superlattice. M myosin, A actin, *a* lattice vector (*i.e.*, centre-to-centre distance between myosin filaments). (**f**) Transverse section of the bare region adjacent to the M-band where the dark-stained thick filaments have a distinctly triangular profile. The interfilament distance ***a*** in (**f**) is about 43 nm.

Once the sliding filament model of muscle contraction had been proposed [1,2], the problem of how muscle contraction takes place became one of determining what makes the actin and myosin filaments in the muscle sarcomere slide past each other (Figure 1a). Hugh Huxley [3] saw projections on the myosin filaments (Figure 1c) and he proposed that these could interact with the adjacent actin filaments to produce force and movement. Huxley [3] showed that the projections were due to myosin molecules which were seen to have a globular region at one end of a long rod. The globular end was later shown to be due to two elongated globular heads (Figure 1d) and it had been shown that these could bind to actin and that they hydrolysed ATP. Huxley [4] then came up with the idea of the myosin heads swinging on actin and then detaching in a kind a rowing movement powered by ATP hydrolysis. These basic ideas were well established by 1969 and have stood the test of time. The sequence of steps in the ATPase cycle was defined by Lymn and Taylor [5]. However, to go further into understanding quite how the heads might interact with actin, how many heads would interact at the same time, how long they would stay attached and what the conformational changes involved in the changing head tilt might be, required many further decades of research and even now are not totally settled.

Among other things Huxley [6,7] had shown that in vertebrate muscle the myosin filaments (Figure 1c) and actin filaments (Figure 1b) form a hexagonal array in the muscle A-band with the actin filaments at the so-called trigonal positions in the hexagonal myosin filament array (Figure 1e), such that one actin filament was surrounded by three myosin filaments. The unit cell of the basic hexagonal lattice (Figure 1e, left) contains one myosin filament. The basic structure of actin filaments (Figure 1b) had been seen by Hanson and Lowy [8] to be like two twisting strings of globular molecules giving a helical structure which was later found to have an axial repeat in vertebrate striated muscles of about 37 nm (Figure 1b) [9]. Associated with the actin filaments are the regulatory proteins tropomyosin and troponin.

**Figure 2:**
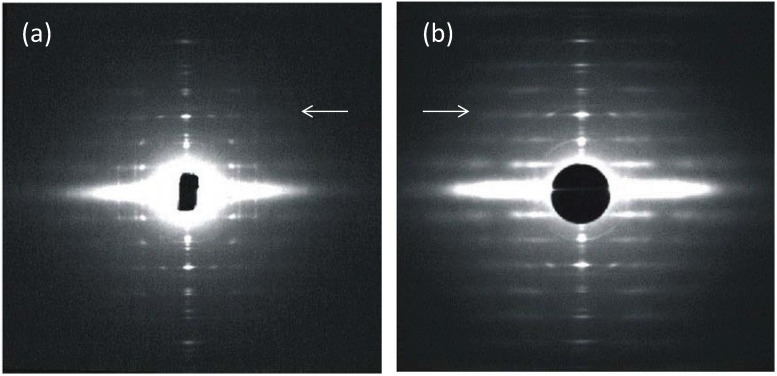

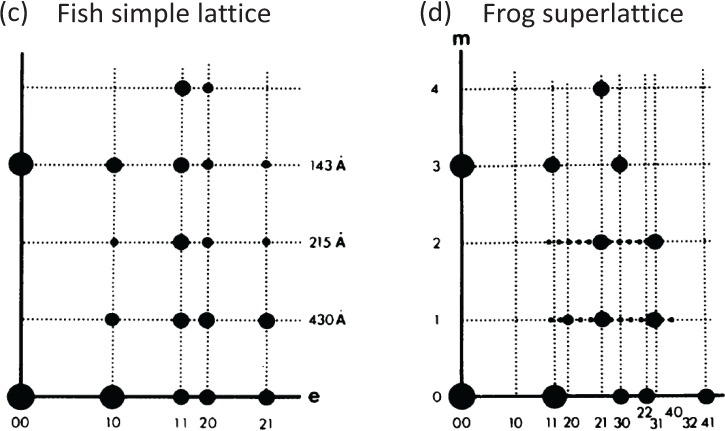
(**a**,**b**) Low-angle X-ray diffraction patterns from fish muscle (**a**) and frog muscle (**b**) (courtesy of Hugh Huxley and Tom Irving), showing the similar layer-line (horizontal line) distribution, but different sampling along the layer lines. The arrows point to the meridional (M3) peak at 14.3 nm. (**c**,**d**) Summary of the X-ray patterns. The sampling in the fish muscle X-ray pattern (**a**,**c**) is very regular (sampling on all layer lines is on the same vertical row lines) indicating the presence of a simple lattice. Sampling in the frog pattern (**b**,**d**) is different on different layer lines and shows a superlattice. (**c**,**d**) adapted from [10] with permission from Elsevier.

An early question to be answered was how were the myosin heads in a relaxed muscle organised around an actin filament and how did this array of heads fit in with the geometry of the actin filaments with which they would interact. The geometry of the myosin head array around actin then depended principally on two things. One was the structure of the myosin filaments themselves; for example their axial repeats and the arrangement of the myosin of heads on the filament surface. The other concerned the relative rotations of the three myosin filaments around the actin; did they all have the same rotation or were the myosin filaments rotated relative to each other either in a random way or in a specific regular structure. In their early work using X-ray diffraction data, a classic *tour de force*, Huxley and Brown [9] clearly showed that the myosin filaments have strong axial repeats of 14.3 nm and 42.9 nm and they suggested a possible helical arrangement of the myosin heads to explain these observed spacings. They also thought they could see evidence of a particular set of rotations between adjacent myosin filaments. This was based on the appearance of apparent sampling along the 42.9 nm X-ray layer line, known to come from the myosin heads, that was different to the sampling on the equator (Figure 2b,d). Huxley and Brown had taken the myosin filament head array to be described by a 2-stranded helix of subunit axial repeat 14.3 nm and true repeat 42.9 nm to explain the observed spacings in their X-ray diffraction patterns from frog muscle. With the advice of Aaron Klug and based on the apparent layer line sampling they then suggested that the hexagonal A-band lattice of 2-stranded myosin filaments contained filaments with three different rotations distributed in a systematic way to give a so-called superlattice structure where the repeating unit cell had a side of ***a***√3 where *a* is the centre-to-centre separation of nearest neighbour myosin filaments (Figure 1e). This larger superlattice unit-cell contains three myosin filaments (Figure 1e, right).

## 2. A Regular Superlattice Was Not Found: Evolution Had Opted for a Disordered Superlattice

Since the superlattice contains three myosin filaments in principle each of these three could be in a different environment. Huxley and Brown [9] supposed, based on a 2-stranded myosin filament, that the rotations between adjacent filaments along the long diagonal of the lattice in Figure 1d right were systematically 0, 60° and 120°. The filament symmetry issue was then addressed by Squire (1971) [11] who showed that the X-ray and other data used by Huxley and Brown could be explained in principle by myosin filaments with either 2-start, 3-start or 4-start helical symmetry. In 1972 Squire then preferred the 3-start interpretation which later proved to be correct [12,13,14,15,16,17]. Squire [13] then showed that a regular superlattice of the right size could be accommodated by 3-stranded filaments if they were systematically rotated by 40° rather than the 60° expected by Huxley and Brown.

**Figure 3:**
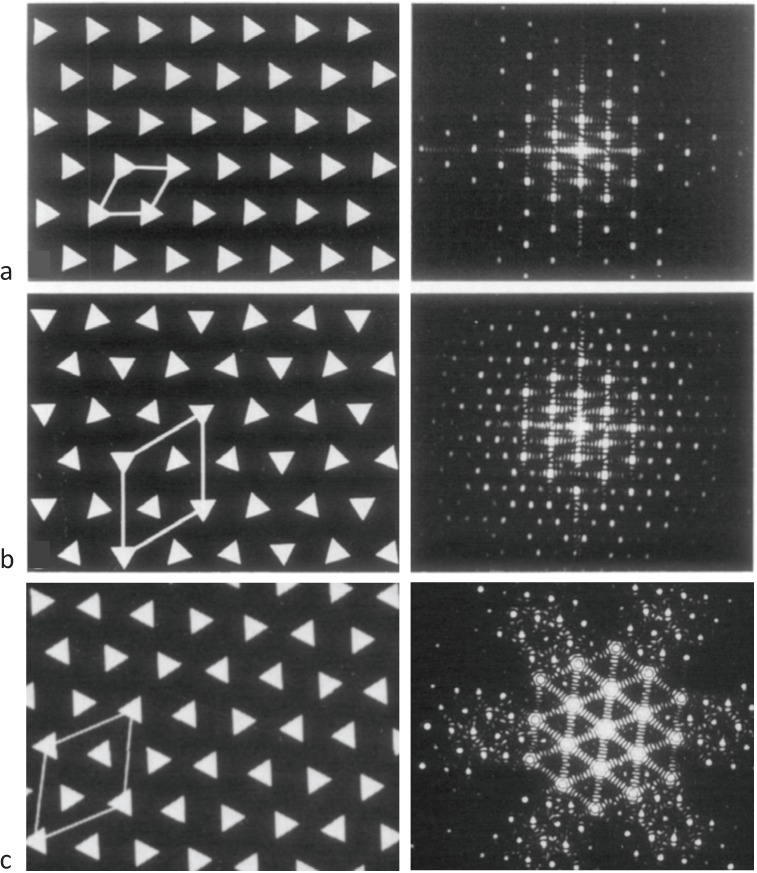
Images of model bare-regions comprising (left) triangular profile filaments on a hexagonal lattice and (right) their diffraction patterns. (**a**) Single orientation filaments as seen in bony fish muscle. (**b**) Perfect superlattice formed by relative rotations of 0, 40° and 80° between adjacent filaments. (**c**) Bare-region model for frog muscle with only two orientations, 0 and 180° (or 60°) apart as observed in electron micrographs and constructed using the no-three-alike rules. The randomness in (**c**) leads to noise in the diffraction pattern in the right panel compared with the ordered noise-free patterns in (**a**) and (**b**). Adapted from [18], with permission from Elsevier.

The form of the superlattice was studied directly by Luther and Squire [18] using a particular feature of vertebrate striated muscle myosin filaments that was very helpful. In the middle of the A-band adjacent myosin filaments are cross-linked by the M-band proteins, myomesin and M-protein (Figure 1a; [14,19,20,21,22,23,24]. The exact nature of this crosslinking depends on the particular muscle and fibre type involved (e.g., slow or fast skeletal or cardiac). But in each case just to the sides of the M-band, in the bare regions, the backbones of the myosin filaments appear triangular in profile. This in itself was a good indicator of the 3-fold rotational symmetry of the filaments. But the distinctive triangular profiles also show the relative rotations of adjacent myosin filaments. Luther and Squire [18] made models of bare region triangle lattices expected from both ordered and disordered superlattice arrangements (Figure 3) and compared them and their diffraction patterns to electron micrographs of carefully prepared frog sartorius muscle. Despite meticulous searches, a regular lattice formed by two or three orientations of the filaments was not found. They eventually found that the myosin filament triangular profiles in frog sartorius muscles have one of only two orientations 60° (or 120°) apart and that these two orientations are distributed in only a quasi-regular manner with a great deal of disorder that locally gave rise to a superlattice unit cell of exactly the size and shape described by Huxley and Brown [9], but with different contents. Because of the disorder, the coherent unit of the lattice was limited to about two superlattice unit cells in any direction instead of the infinite lattice expected from perfectly ordered superlattices. In other words although a perfect superlattice with infinite order based on three unique orientations of the filaments was possible, evolution opted for a much less ordered superlattice based on two orientations.

## 3. The Nature of the Superlattice: The “No-Three-Alike” Rules

**Figure 4:**
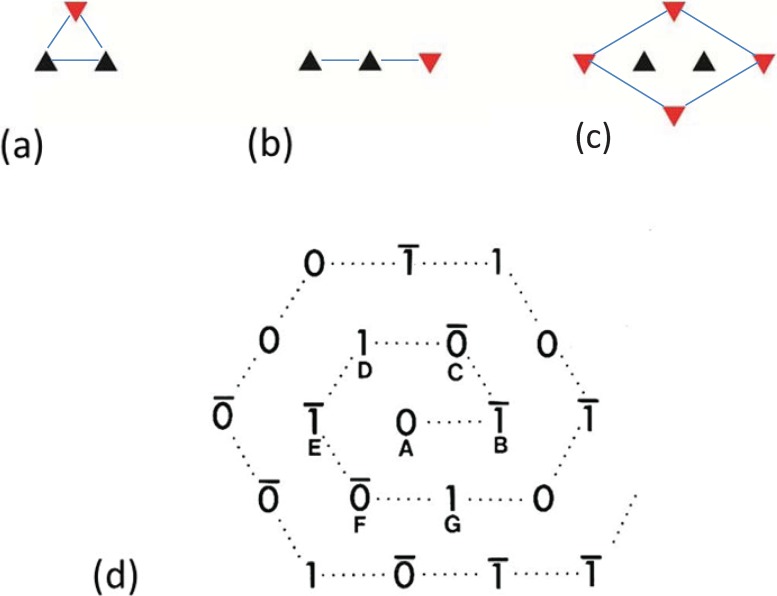
Illustrations of the applications of the no-three-alike rules. (**a**) Rule 1: Three adjacent filaments forming a triangle cannot all have the same orientation. (**b**) Rule 2: Three filaments along a row of the hexagonal lattice cannot all have the same orientation. (**c**) Applying these two rules gives rise to a superlattice unit cell. (**d**) Generation of a synthetic bare region. Symbols 0 and 1 represent the two myosin filament rotations (0° and 60°). The lattice was generated by travelling along a spiral from the centre A. A bar above the lattice position indicates that the orientation was not defined by the adjacent filaments and so was determined by tossing a coin. (From [18], with permission from Elsevier).

**Figure 5:**
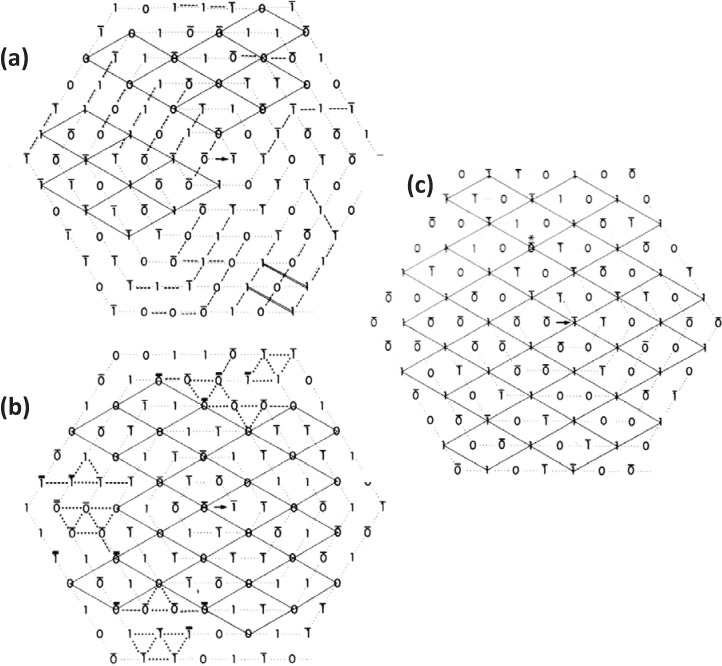
Generation of synthetic bare region model lattices using the no-three-alike rules: (**a**) rule 1 alone; (**b**) rule 2 alone; and (**c**) using rules 1 and 2 together. The lattices were generated in a spiral fashion as in Figure 4d. Superlattice unit cells are outlined by solid lines. Rule breaking is indicated by dotted and dashed lines. Filament orientations not determined by the rules have a line over the symbol. Filaments with forced breaking of the rules have a thick or double line over. (**c**) Shows more superlattice extent than (**a**) or (**b**), but even here the unit cell contents (*i.e.*, the rotations of the filaments in the middle of the unit cells) vary across the lattice; there isn’t a regular structure. (Adapted from [18], with permission from Elsevier).

By analysing the distribution of filament rotations in electron micrographs of the frog sartorius muscle bare region across particular A-bands it was noticed by Luther and Squire (1980) [18] that rarely did three neighbouring myosin filaments at the corners of a triangle have the same rotation. If any two had the same rotation, the third was almost always of the opposite rotation (Figure 4a). In addition, if two myosin filaments in a line had the same rotation, then the third filament along the line tended to have the opposite rotation (Figure 4b). We called these the “no-three-alike” rules 1 and 2 respectively. Applied together these two rules could generate a single perfectly ordered superlattice unit cell (Figure 4c). Larger synthetic A-bands were then generated using these two rules to see what would happen (Figure 4d and Figure 5). For simplicity the synthetic lattices were grown in a spiral fashion (Figure 4d). In some cases when building the next myosin filament into the lattice the rotation of this filament was not determined by the rotations of the adjacent filaments. In this case the filament rotation was determined by tossing a coin (indicated by a bar above the symbols in Figure 4d). The result of this procedure (Figure 5) was to produce a superlattice array of the same size and shape as that observed in frog sartorius muscle, but with variable unit cell contents, extents and degrees of disorder.

## 4. Not All Vertebrate Striated Muscles Have a Superlattice Structure

At the same time as analysing the bare regions of frog sartorius muscle A-bands [18], Luther *et al.* [15] studied the bare regions of some bony fish muscles. Electron micrographs of thin transverse sections of killifish muscles had previously been obtained by Pepe (1975) [25]. These suggested that the triangular profiles in the bare region all had roughly the same rotation. Further careful analysis by Luther *et al*. [15] confirmed this result in another bony fish, the roach; it appeared that all the myosin filaments within a given A-band in these bony fish muscles have exactly the same rotations and that there is, therefore, a simple lattice of myosin filaments not a superlattice (Figure 1e (left), Figure 2a,c and Figure 3a).

## 5. Evolution of Simple and Superlattice A-Bands

**Figure 6:**
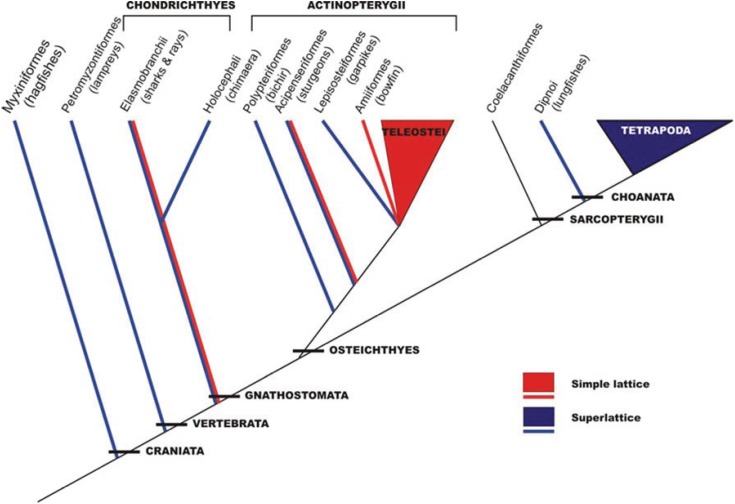
Phylogeny of craniates showing the interrelationships of the major groups and the distribution of the two lattice types. The species studied is enclosed within brackets. Several species were studied in the teleostei (roach, plaice, black molly) and tetrapoda (turtle, frog, rat, rabbit, chicken, human) classes. (Adapted from [26], with permission from Wiley).

**Figure 7:**
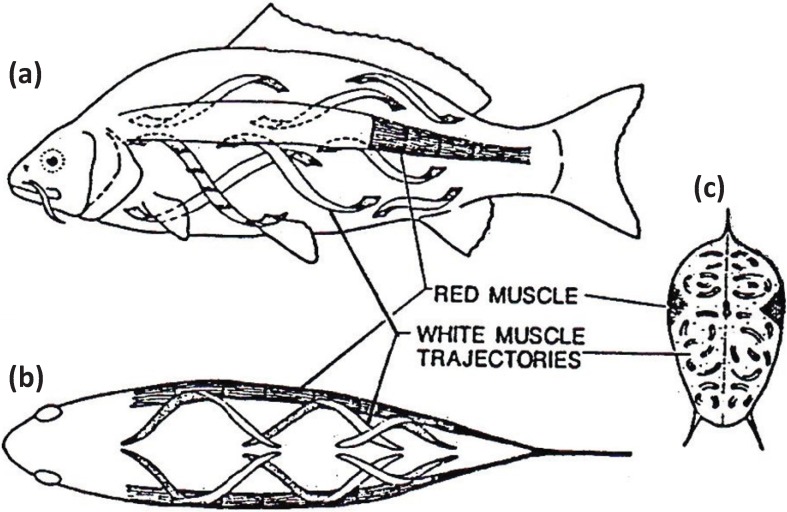
Muscular anatomy of the carp *Cyprinus carpio* in (**a**) longitudinal view, (**b**) dorsal view and (**c**) cross-sectional view. The red muscle (at the lateral line) forms a thin sheet just under the skin and extends to a depth of about 10% of the distance to the backbone. The red muscle cross-section is exaggerated here for illustrative purposes. The white fibres in the body of the fish run helically rather than parallel to the long axis of the fish. According to calculations by Alexander [27] they shorten by only about a quarter as much as the red muscles for a given curvature of the body. From [28], reproduced with permission from Macmillan Publishers Ltd: (Nature).

This analysis in the early 1980s showed that vertebrate striated muscles had one of two basic types of A-band lattice, the simple lattice in some bony fish and the superlattice in muscles like those of the frog. Luther *et al*. [26] then carried out a survey of A-band types across the vertebrates; were these the only two kinds of vertebrate A-bands and if so how were they distributed among different animals? The result is shown in Figure 6. No kinds of A-band order other than simple lattice and superlattice were found. However, it can be seen in Figure 6 that while all the higher vertebrates (mammals, including humans, reptiles, birds, amphibians; *i.e.*, all the tetrapods) have superlattice muscles, the distribution of simple and superlattices in earlier forms is more complicated. All the teleosts (bony fish) so far studied appear to show the simple lattice. On the other hand, the very early forms, hagfish and lamprey for example, have superlattice muscles, so the superlattice appears to have developed first. Sharks and rays are unusual in having both A-band types; their skeletal (white) muscles have the superlattice, whereas the red muscles (lateral line; Figure 7) have the simple lattice. This may also occur in other primitive fish like sturgeon.

## 6. Origins of Different A-Band Lattices: Interactions at the M-Band

**Figure 8:**
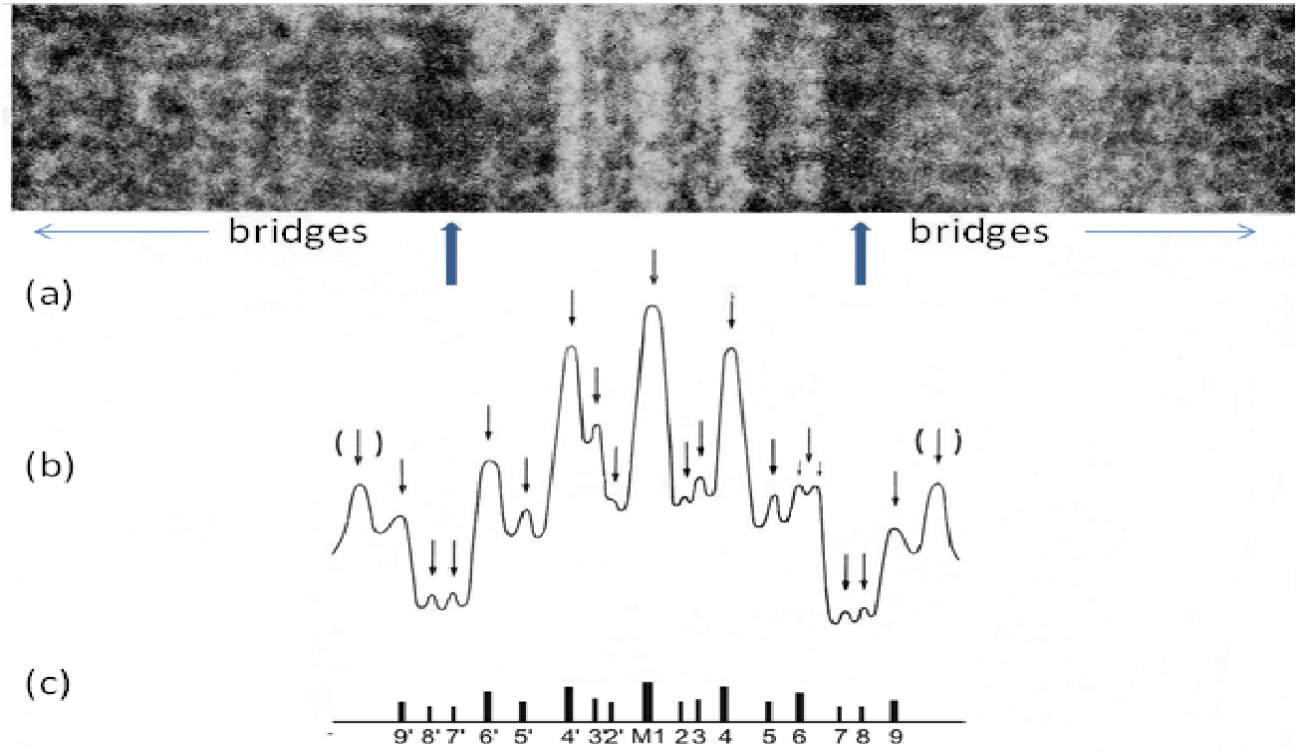
(**a**) Enlarged region of a longitudinal cryo-section of the A-band in human *m. tibialis anterior* showing the central M-band lines, the flanking bare regions (strong arrows) and the starts of the two bridge regions. Protein is white in this figure. (**b**) Density profile through the middle of the micrograph in (**a**) showing the prominent M-bridge densities. The main M-bridges are labelled as in (**c**). (Adapted from [20] with permission from Elsevier).

The teleosts and tetrapods are by far the dominant vertebrate groups with 23,000 and 21,500 species, respectively, but they have different A-band lattices which presumably provided them with evolutionary advantages. In thinking about what might cause the A-bands in different muscles to be different it became apparent that the only structures known about that might define these lattices were the M-band proteins providing cross-links between neighbouring myosin filaments (Figure 1a and Figure 8). From the work of Sjostrom and Squire [20] and Pask *et al.* [29] a number of different M-band appearances had been seen in longitudinal sections of different striated muscles depending on their fibre type. In particular the M-band showed up to five very dense lines of protein symmetrically placed around the middle of the M-band (Figure 8). In addition to a central line of density called M1 there were lines about 22 nm on each side of M1 which were labelled M4 and M4’ and another pair of lines about 44 nm from M1 which were called M6 and M6’. Weaker densities were seen at lines labelled M2, M3 and M5 and their symmetrically related positions. Even in muscles which all showed the same superlattice A-band, some M-bands had M1, M4, M4’, M6, M6’ all strong and were termed 5-line M-bands and other M-bands had just M1 and M4, M4’ strong (the fastest fibres); 3-line M-bands. Yet other M-bands (slow and some cardiac muscles) were like a 5 line M-band except that M1 itself was missing; a 4-line M-band. In other words the M1 and M6 lines appeared variable, whereas M4 and M4’ were always present. So whatever defines the A-band simple and superlattices must presumably involve interactions at M4.

**Figure 9:**
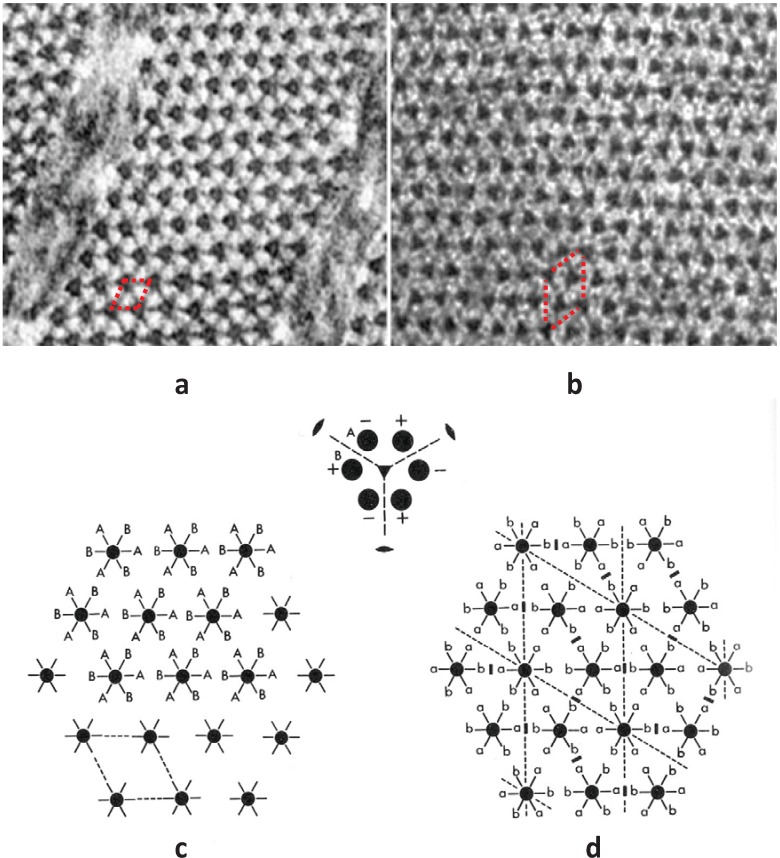
(**a**,**b**) Electron micrographs of muscle cross-sections close to the M-band, in (**a**) bony fish (plaice) (**b**) frog muscle. (**c**,**d**) M-band interactions using (**c**) unlike interactions (A to B); and (**d**) maximising like interactions (a to a and b to b); a superlattice is formed. (From [18], with permission from Elsevier).

**Figure 10:**
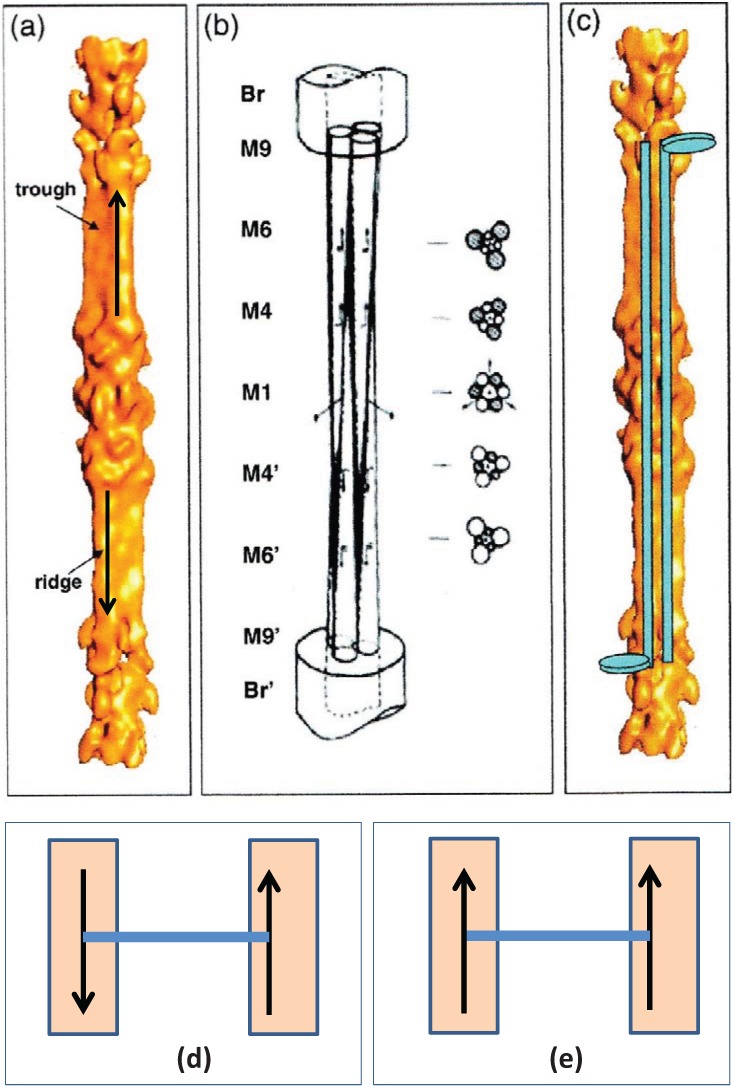
(**a**) 3D reconstruction of the myosin filament bare zone and its interpretation: (**b**) in terms of three overlapping conical rod shaped subfilaments; and (**c**) in terms of antiparallel groups of myosin rods. (From [30] with permission from Elsevier). (**d**) Up to down interaction at M4 as in bony fish muscle; (**e**) up to up or down to down interactions at M4 as in superlattice muscles.

An understanding of what M4 might be doing then depended on some structural knowledge of the myosin filament. Vertebrate striated muscle myosin filaments are aggregates of myosin molecules (Figure 1c,d; [3]). The myosin molecules appear as rods with two heads at one end (Figure 1d). The myosin rods were thought by Huxley to be packed antiparallel in the middle of the myosin filament and parallel to each other along the two ends of the filament giving what is called a bipolar structure. Analysis by Luther *et al*. [15] of myosin filament cross-sectional profiles through the A-band then showed that the two halves of a given myosin filament were related by what is termed 32 point group symmetry (central drawing in Figure 9). This means that in any cross-section the filaments have 3-fold rotational symmetry; rotating them around their long axis by 120° leaves them unchanged. It also means that in a plane perpendicular to the filament long axis and at the level of M1 in the middle of the M-band there are three 2-fold rotation axes; flipping the whole filament by 180° around one of these axes leaves the filament unchanged. This 32 point group structure was later confirmed by electron microscopy and single particle analysis of the middle part of isolated vertebrate myosin filaments [30], as shown in Figure 10.

Using Figure 10a, if we think of a myosin rod with its heads above the M-band to be an “up” molecule (up arrow in Figure), then those myosin molecules with heads below the M-band can be termed “down” molecules (down arrow). At the level of M1 the myosin filament backbone therefore consists of three sets of “up” molecules alternating with three sets of “down” molecules (Figure 10b,c). Likewise at a particular M4 level, three sets of myosin rods will be “up” and three sets “down”. But the myosin filament interacts with six nearest neighbours. The question then arises whether M-bridges linking to “up” molecules at M4 on one filament interact at the other end with myosin rods that are “up” or “down”. It turns out that if all “up” myosin molecules always link to “down” molecules in the neighbouring filament (Figure 10d), and *vice versa*, the simple lattice is always produced. This is like saying that a corner of a triangular profile of a filament in the bare region always points to a triangle side in the neighbouring filament (Figure 9a). All such “up” to “down” (or point to side) interactions can be satisfied and the resulting lattice is a completely regular simple lattice. It is equivalent to saying that the interactions are from A on one filament to B on its neighbour (Figure 9c). B here is just A turned upside down.

What happens if, alternatively, M-bridges want to interact with “up” myosin rods at both ends, or “down” myosin rods at both ends (Figure 10e)? In this case (Figure 9d) the lattice cannot be regular; the preferred interactions are here shown as from “a” to “a” or “b” to “b”. There are occasions where there is ambiguity about how a filament should be placed in the lattice. In fact a disordered superlattice is automatically generated just as observed by [18]. The conclusion is that different interactions at the ends of M-bridges at level M4 may be sufficient to define whether a muscle has a simple lattice or a superlattice A-band. One can speculate that different isoforms of myosin or of the M4 M-band protein myomesin or even of the M-band part of titin might be sufficient to do this [22,23,24,31].

## 7. The Distributions of Myosin Heads in Different A-Band Lattices: Does the Lattice Type Make a Difference?

We have seen that there are two different kinds of vertebrate A-band lattice and we have found how the two different lattices might arise. We have also seen a particular pattern of evolution of the two lattices across the vertebrate kingdom (Figure 6). Luther and Squire [18], Luther *et al*. [15] and Harford and Squire [10] thought it was likely that the triangular profiles in the bare region were directly related to the 3-start helices of heads in the rest of the A-band and that the myosin heads were likely to be organised into the same filament lattices (simple and super) as indicated by the bare region analysis. They confirmed that the regular simple lattice bare region structure in bony fish muscle was also evident in the myosin crossbridge array because the X-ray layer lines at 42.9 nm and 14.3 nm (Figure 2a,c) produced by the head arrays in bony fish muscle were beautifully sampled by the same simple lattice structure. On the other hand, the same layer lines from frog muscles (Figure 2b,d); Huxley and Brown [9] were sampled in a much more complicated and incomplete way as would be produced by a typical disordered superlattice (as shown in Squire [32]; Figure 7.27). In summary the two different A-band lattices of 3-start myosin filaments produce different arrangements of the myosin heads around an actin filament; a very regular myosin head array in simple lattice muscles and a more complicated irregular array in superlattice muscles. The question then arises what difference this might make to the way in which the heads can interact with actin.

**Figure 11:**
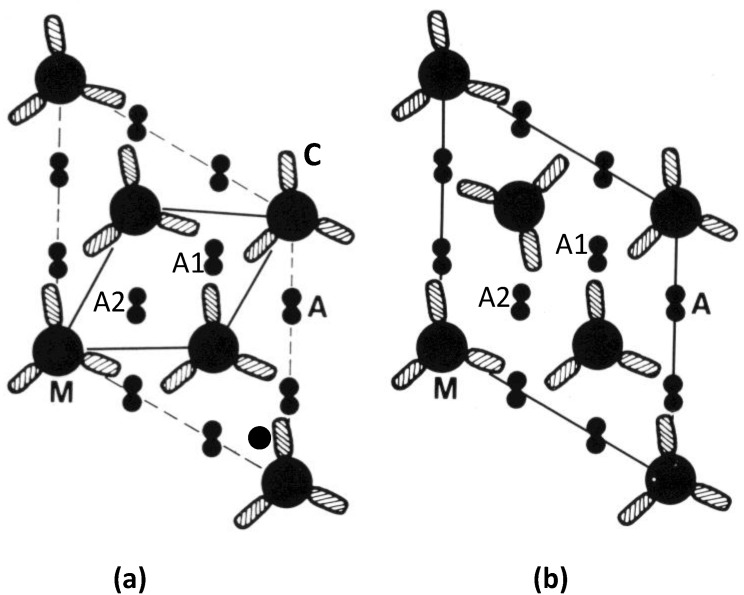
Actin (A) environments in the simple lattice (**a**), and superlattice (**b**), at one crown level along the myosin filaments (M). Each cross-hatched oval shape (C) represents the two myosin heads on each myosin molecule (*cf*. Figure 1d). The superlattice in (**b**) has the four heads of two myosin molecules approaching actin filament A1 at this level, and the two heads of one molecule approaching A2. In (**a**), from bony fish muscle, six heads from three myosin molecules approach A1 at this level and no heads approach A2. The distribution varies on the other two crowns in a 42.9 nm axial repeat of the myosin filaments, but the same kinds of effects occur. For example filament A2 in (**a**) has heads interacting with it at other levels through the A-band. (Adpated from [10] with permission from Elsevier).

Figure 11 compares the actin environments in the two kinds of A-band lattice at one of the myosin head crown levels. It can be seen that in the simple lattice (a) at this particular level some actin filaments, labelled A1, have the heads of three myosin molecules pointing towards them at the same level, whereas actin filaments like that labelled A2 have no heads pointing towards them at that level. The effect of the superlattice filament arrangement is different (Figure 11b). Here the heads of just two myosin molecules can interact with actin filament A1 and the heads of one molecule can interact with actin filament A2 at that level. So in simple lattice muscles there will be six heads trying to interact with a short region of filament A1 at the same time on this level, whereas there are at most four heads trying to interact with A1 in a superlattice muscle. We discuss two possible consequences of this. There may well be others.

Firstly, it has been found that the orientation of the actin monomers adjacent to the myosin heads has a strong effect on how easily the heads can bind to that particular monomer. Successive actin monomers move azimuthally towards and then away from the viewer as the viewer moves axially along the filament (or as the actin filament slides past) in Figure 1b and a myosin head next to such a filament will “see” the same thing. Since the strong interaction of myosin heads with actin is stereospecific (*i.e.*, it has a defined 3D geometry), it will be easier for the head to interact with actin monomers at some positions along the actin filament than at others. Reedy [33], in his pioneering studies of insect flight muscle, originally came up with the idea of actin target areas or zones where heads prefer to attach. However, at the time, he thought the insect myosin filaments to be 2-stranded, to have four heads at each 14.5 nm-spaced crown of heads on each filament and that the beautifully organised lattice and matched axial repeats of the actin and myosin filaments in insect flight muscle allowed matching of the arrangement of heads to the arrangement of actin sites, so that in rigor all heads could attach to actin. After reconsidering the rotational symmetries of different myosin filaments, Squire [11] thought that insect flight muscle myosin filaments might have more than two strands of heads; he thought at the time there might be up to six strands. Then in 1972 Squire [12] showed that the orientation of actin subunits in rigor insect flight muscle could be such that some of the heads on a multi-stranded myosin filament simply could not attach to actin because of the steric constraints discussed above. In fact it was shown later that the number of strands is actually four in insect flight muscle myosin filaments [34], but the idea that local actin geometry might make some heads totally unable to attach to actin at particular filament overlaps had been established. This idea was supported when the number of heads attached in rigor muscles was found to be about 100% in vertebrate skeletal muscles, but only about 80% in insect flight muscle [35]. The presence of geometrically determined target areas on actin was later confirmed by Steffen [36] using optical trap methods.

With the idea of actin target areas in mind, the presence of six heads at one axial level, as in filament A1 in the simple lattice (Figure 11a), might mean, whatever the axial position of the actin filaments relative to the myosin crowns, that as the sarcomere length changes it would always be easy for some of those myosin heads at that level to attach to actin. However, on this basis, some heads at the same level might be totally unable to attach to actin because of the heads that are already blocking the available sites; a smaller total number of heads might attach.

The second possibility builds on this last point and supposes that the more even distribution of heads along actin that occurs with the superlattice muscles might mean that on average more heads can attach at any one time than in simple lattice muscles, and that filament sliding is less jerky than it might be in fish muscle. A consequence of this might be that superlattice muscles can produce more tension than simple lattice muscles. We have tested this idea in a very preliminary way by doing a quick survey of the published literature on maximum forces per unit area in a variety of muscles. What we have found so far is that there do appear to be differences in the average forces per unit area between superlattice muscles where the forces are relatively high (roughly 180 kN/m^2^ to 450 kN/m^2^), and simple lattice muscles where the forces are generally lower (roughly 70 kN/m^2^ to 280 kN/m^2^). This requires confirmation and full details will be published elsewhere.

Since simple lattice muscles occur mainly in the bony fish, but not in some cartilaginous fish (Figure 5), and since it appears that superlattice A-bands evolved first, it is pertinent to ask why simple lattice A-bands are advantageous for bony fishes. Two factors come to mind. One is that the bulk of the bony fish is actually the body muscle which defines the streamline shape of the fish. It is not these muscles which are used mainly for slow swimming, but rather the muscles on the lateral line (Figure 7), which are often red, slightly slower muscles than the body muscles. Perhaps it is economical for the fish to have fewer heads cycling on actin on average in the body muscles during normal slow swimming to reduce the general base level of metabolism of ATP in these muscles. In addition, since a main safety strategy of these fish is the escape response which is mainly powered by the white body muscles, perhaps it is advantageous to always have heads that can attach to actin (the six heads near the A1 actin filament), whatever the current sarcomere length, to give maximum escape speed in a very rapid but short burst. As we have seen earlier, it may also be advantageous for superlattice muscles to have their heads more evenly spread along the actin filament to give smoother contractions, and to create higher forces.

## 8. Structural Biology of Vertebrate Striated Muscles: Simple Lattices are Advantageous

One of the reasons why we became so interested in looking at the structure of bony fish muscle is that, in principle, the low-angle X-ray diffraction patterns from vertebrate muscles (e.g., Figure 2a) ought to carry sufficient information for one to define the changing structure in the sarcomere as muscle contraction occurs; particularly the motions of the myosin heads. Much of the early work on this was carried out by Huxley and his collaborators [9,37] and by Elliott and his collaborators [38,39] (see [40] for details) on frog muscles and many fundamentally useful insights were obtained. However, in order to generate realistic models of sarcomere proteins during contraction, in other words to get optimised model fits to the observed intensities, the presence of the disordered superlattice in these muscles and the consequent partial sampling to the myosin layer lines in such diffraction patterns posed a real problem. (However Oshima *et al*. (2012) [41] attempt to remove the partial sample in a recent study). On the other hand, the simple sampling of the fish muscle low-angle patterns (Figure 2a) was much easier to handle (e.g., [42,43]. In addition, the presence of the simple lattice meant that useful image processing and averaging could be carried out on electron micrographs of cross-sections through the A-band to reveal details of filament structure and organisation [15,21,44]. So, as well as fish muscle being interesting in its own right, the presence of the quasi-crystalline simple lattice A-band structure has been very helpful indeed as a tool for structural biology purposes.

One thing to bear in mind, though, is that a very great deal of useful X-ray diffraction information has been obtained already from frog muscles. It would therefore be a very good thing to be able to model diffraction data from frog muscles in an objective fashion as has been done for simple lattice muscles [42,43]. Since, through the work of Millane and his collaborators [45], we now know the statistics of the superlattice in a number of muscles, it is possible in principle to include the observed kinds of superlattice disorder in analysis of the low angle X-ray diffraction data from frog muscles. An approximate attempt at dealing with the superlattice was made by Koubassova *et al*. [46,47]. However, a new approach based properly on observed superlattice statistics has been utilised in a more rigorous analysis by Wojtas *et al*. [48], who automatically generated the correct “sampling” on different layer lines as observed, and further work is in hand.

## 9. Conclusions

The purpose of the myosin filaments in muscle is to interact with the actin filaments to generate shortening forces. The M-band assembly tethers the myosin filaments in a hexagonal lattice to facilitate the interaction with actin. In addition, the M-band determines the orientation of the myosin filaments; the M-band determines the head environment around an actin filament. Evolution appears to have opted for the less ordered superlattice arrangement of the myosin filaments from primordial times starting with the craniates (hagfish) and earliest vertebrates (lamprey) up to most of the later vertebrates. Presumably the superlattice arrangement confers an advantage in the majority of vertebrates including tetrapods. The simple lattice form, appearing later in evolution, must presumably confer an advantage to the lifestyle of the teleosts.

The intriguing twist to this story is the muscle organisation in cartilaginous fish. Here, the slow and fast muscles have distinct lattice forms, simple and super, respectively. This shows the importance of the lattice form for the function of the muscle. We have suggested here that there are differences in the force levels reported for different muscle types, but more analysis is needed and a clear cut conclusion cannot be made so far. Further work will need to be done to understand the advantage of superlattice muscles for most of the vertebrates and for simple lattices for the teleosts.

The story of the discovery and analysis of the superlattice underlines the synergy of X-ray diffraction and electron microscopy in understanding the fine structure of biological samples. The X-ray diffraction spots observed by Hugh Huxley showed clearly that a superlattice of a certain size was present. To understand the nature of the superlattice, direct imaging was required as we reported eventually in Luther and Squire [18]. In fact, this kind of synergy may have helped the development of electron microscopy of biological samples. X-ray diffraction from living muscles showed the presence of particular periodicities that can be seen in electron micrographs of the same muscle if the fixation and staining of the muscle are good enough [49]. X-ray diffraction can therefore be used to monitor the quality of electron microscopy data. At the same time, X-ray diffraction does not give an image of the diffracting object. Analysis of electron micrographs can aid in the interpretation of the X-ray diffraction patterns. The combination of the two techniques, as is possible in the case of muscle, is extremely powerful.
